# Social information use in adolescents with conduct problems and varying levels of callous‐unemotional traits

**DOI:** 10.1002/jcv2.12067

**Published:** 2022-03-05

**Authors:** Anne Gaule, Leonardo Bevilacqua, Lucas Molleman, Ruth Roberts, Anna C. van Duijvenvoorde, Wouter van den Bos, Eamon J. McCrory, Essi Viding

**Affiliations:** ^1^ Division of Psychology and Language Sciences University College London London UK; ^2^ Department of Psychology and Human Development, Institute of Education UCL, London, UK; ^3^ Amsterdam Brain and Cognition University of Amsterdam Amsterdam The Netherlands; ^4^ Department of Developmental Psychology University of Amsterdam Amsterdam The Netherlands; ^5^ Social Psychology Tilburg University, Tilburg, The Netherlands; ^6^ Department of Developmental and Educational Psychology Leiden University Leiden The Netherlands; ^7^ Leiden Institute for Brain and Cognition Leiden University Leiden The Netherlands

**Keywords:** callous‐unemotional traits, conduct problems, social cognition

## Abstract

**Background:**

Adolescents with conduct problems (CP) are characterised by difficulties with social relationships and display atypical social cognition, such as when interpreting emotional expressions or engaging in social problem‐solving. One important aspect of social cognition that warrants investigation is the degree to which these adolescents factor others' views into their already held beliefs, and strategies used to do so. Effective social information use enables attunement to social environment, cooperation, and social problem‐solving. Difficulties in this regard could contribute to problems in social interactions in adolescents with CP, and may vary with adolescents' high (CP/HCU) versus low levels of callous‐unemotional traits (CP/LCU).

**Methods:**

We compared social information use in boys (11–16 years) with CP/HCU (*n* = 32), CP/LCU (*n* = 31) and typically developing (TD) peers (*n* = 45), matched for IQ. Participants provided estimates of numbers of animals on a screen, saw another adolescent's estimate, and could adjust their initial estimate. We compared two aspects of social information use: (1) degree of adjustment of initial estimate towards another's estimate and (2) strategy use when adjusting estimates.

**Results:**

Degree of adjustment towards another's estimate did not vary across groups, but strategy use did. Adolescents with CP/LCU compromised less following social information than TD peers.

**Conclusions:**

Findings suggest that while adolescents with CP are able to take social information into account, those with CP/LCU use this information in a way that differs from other groups and could be less efficient. This warrants further systematic investigation as it could represent a target for behaviour management strategies. Overall, this study highlights the need for more research delineating the social‐cognitive profile of adolescents with CP/LCU.

## Graphical abstract

Adolescents with Conduct Problems (CP) are characterised by antisocial behaviour and difficulty with social relationships, and their presentation can vary depending on whether they have high (CP/HCU) vs low levels of callous‐unemotional traits (CP/LCU). This study examines social information use (degree of adjustment of beliefs in response to social information and strategy used to do so) in adolescents with CP/HCU, CP/LCU, and typically developing (TD) peers. While all groups adjusted beliefs in response to social information to the same degree as TD adolescents, CP/LCU adolescents used fewer compromising strategies when doing so. This finding provides a potential explanation for social difficulties in children with CP/LCU and suggests avenues for future research that have the potential to inform behaviour management for this group. It also adds to the evidence base indicating heterogeneity among children with CP.

**Key points**

- Adolescents with Conduct Problems (CP) are characterised by antisocial behaviour and difficulty with social relationships. Their presentation can vary depending on whether they have high (CP/HCU) versus low levels of callous‐unemotional traits (CP/LCU)
- This is the first study to examine social information use (degree of adjustment of beliefs in response to social information and strategy used to do so) in adolescents with CP/HCU, CP/LCU, and typically developing (TD) peers
- While all groups adjusted beliefs in response to social information to the same degree as TD adolescents, CP/LCU adolescents used fewer compromising strategies
- This finding provides a potential explanation for social difficulties in children with CP/LCU and suggests avenues for future research that have the potential to inform behaviour management for this group. This finding also adds to the evidence base indicating heterogeneity among children with CP

## INTRODUCTION

Conduct problems (CP) are one of the leading causes of referral to mental health services during childhood and adolescence (National Institute for Health and Care Excellence, 2013), and incur large individual and societal costs (Richards et al., 2009). The behaviour of young people with CP violates the rights of others and/or age appropriate norms (American Psychiatric Association, 2013). Adolescents with CP have difficulties with social relationships, are likely to experience social rejection, and to have lower social competence compared to peers (Dodge et al., 1986; Loeber & Farrington, 2001; Webster‐Stratton & Lindsay, 1999). They demonstrate difficulties with social problem‐solving, conflict‐management and collaborative play—often relying on aggressive or coercive strategies (Dodge et al., 1986; Ladd, 1990).

Research into how adolescents with CP process social information can help to elucidate why they display antisocial behaviour and often demonstrate social difficulties. The influential Social Information Processing (SIP) model (Crick & Dodge, 1994) posits that aggressive responses to social stimuli occur as a result of cognitive processing biases or deficiencies over a sequence of steps that starts with cue encoding and finishes with response enactment (Crick & Dodge, 1994). The most prominent finding stemming from SIP research is the tendency of children and adolescents with CP to interpret ambiguous social cues as aggressive (‘hostile attribution bias’; Verhoef et al., 2019). Studies have also reported that aggressive behaviour is linked to both generation of atypical social responses and atypical evaluation of social responses when considering hypothetical scenarios in clinically referred (de Castro et al., 2005) and non‐referred (Dodge et al., 1986) samples of children and adolescents.

A number of recent studies have focused on how different aspects of social‐cognitive processing may differ between subgroups of adolescents with CP and potentially explain their varied pattern of social difficulties. High versus low levels of callous‐unemotional (CU) traits (including lack of remorse and empathy) are one way of subgrouping adolescents with CP (CP/HCU vs. CP/LCU; Frick et al., 2014). Extant data suggests that partially divergent social‐cognitive profiles may underlie antisocial behaviour and social difficulties in these groups. Adolescents with CP/HCU appear to place less importance on social affiliation than CP/LCU and typically developing (TD) peers (Blair et al., 2014; Viding & McCrory, 2019), whereas adolescents with CP/LCU may be less flexible and more aggressive when confronted with social problems than CP/HCU and TD peers (Blair et al., 2014; Frick et al., 2014; Waschbusch et al., 2007).

Specifically, adolescents with CP/HCU appear to be less responsive to others' distress, and demonstrate a lower propensity for social affiliation than TD peers and those with CP/LCU, perhaps driven by a lower responsiveness to positive affiliative cues (Blair et al., 2014; Hodsoll et al., 2014; O’Nions et al., 2017; Viding & McCrory, 2019; Waller & Wagner, 2019). They also demonstrate atypical evaluation of their own behavioural responses and appear to value non‐typical social goals. For example, high CU traits have been associated with increased expectations that aggressive behaviour will produce positive consequences (Pardini et al., 2003) and a higher likelihood of endorsing social goals associated with respect, revenge, and dominance in mixed gender samples of adjudicated adolescents (Pardini, 2011). Additionally, adolescents with CP/HCU show reduced prosocial behaviour relative to CP/LCU and TD peers (in a clinical sample of adolescent males; Sakai et al., 2016). However, adjudicated adolescents with CP/HCU do appear to have good understanding of the social consequences of their aggressive behaviour (Pardini, 2011) and children with CP/HCU (in a predominantly male clinical sample aged 7–9 years) demonstrate good understanding of others' intentions, at least when affect is not involved (Anastassiou‐Hadjicharalambous & Warden, 2008). Thus, research indicates that adolescents with CP/HCU may possess typical social understanding, but prioritise their own goals in social situations—perhaps due to their reduced propensity to empathise and affiliate with others (Haas et al., 2018). This might contribute to their particularly serious antisocial behaviour and impoverished social relationships.

In contrast to those with CP/HCU, adolescents with CP/LCU appear capable of feeling guilt and empathy, and tend to aggress when there are environmental triggers such as a perceived threat (Frick & Morris, 2004; Frick & Viding, 2009). Findings related to social cognition with this group are less clear, as the majority of social‐cognitive research has focussed on CP/HCU or CP in undifferentiated samples. However, some evidence suggests that adolescents with CP/LCU may demonstrate atypical social understanding relative to CP/HCU peers. Waschbusch et al. (2007), in a (predominantly male and clinically referred) sample of children aged 7–12, found that CP accompanied with low levels of CU traits was associated with more overtly aggressive, less prosocial, and less flexible and relevant social problem‐solving solutions than those of their peers with CP and higher levels of CU traits. However, the behavioural evidence‐base is still relatively limited in terms of tasks utilised to date, as well as studies actively comparing CP/HCU and CP/LCU with TD adolescents.

To our knowledge, social information *use—*here defined as the degree to which feedback from others is incorporated into beliefs and the strategies used to do this—has yet to be examined in adolescents with CP. Social information is critical in shaping and guiding decision‐making and behaviour, providing crucial inputs for a range of social‐cognitive processes. We rely on social feedback to infer whether others approve of our decisions, choices, and behaviours (Cialdini & Goldstein, 2004). Effective use of social information allows us to learn about successful behavioural strategies while avoiding costly individual trial‐and‐error (Boyd & Richerson, 1985; Kendal et al., 2018). It also enables individuals to attune to their social environment, facilitating cooperation and coordination with social partners (Boyd & Richerson, 1985; Sigmund et al., 2010; Surowiecki, 2005). Conversely, atypical social information use may hamper the formation and maintenance of social relationships. Investigating the degree to which adolescents with CP use social information (in the form of feedback from others) to adjust their judgments, as well as the strategies they use to do so, may shed more light on the cognitive mechanisms underlying social difficulties commonly observed in this group.

Studies examining this form of social information use typically employ belief updating paradigms: participants make a judgment or an estimate, receive information about another's judgment or estimate, and can then update their initial response if they choose. In these paradigms, updating one's initial estimate in response to information from others implies that a person perceives that using such information will improve their accuracy on the task—thereby improving their likelihood of winning points. Studies using this design indicate that TD adolescents use social information to a greater degree than adults (Costanzo & Shaw, 1966; Knoll et al., 2015). This may be because adolescence is a sensitive developmental period, where young people are increasingly independent and tend to make decisions in pursuit of social acceptance (Gardner & Steinberg, 2005; Knoll et al., 2015). Research has also shown that adolescents frequently adopt relatively simple ‘all‐or‐nothing’ adjustment strategies to incorporate social information into their existing beliefs (copying social information or sticking with original estimates), rather than more complex integrative ‘compromising’ strategies (taking a weighted average of original estimates and social information; Molleman, Kanngiesser, & van den Bos, 2019). It is important, however, to consider that there may be individual differences among adolescents. Prior research has not addressed whether this form of social information use varies in adolescents with CP or in relation to CU traits. The present study, therefore, aims to shed light on how this form of social information use looks in these groups and whether this differs from TD adolescents.

CP/HCU, CP/LCU and TD participants performed a task where they were asked to estimate the number of animals shown in an image and could then adjust their estimate after observing social information (another participant's estimate). This allowed us to examine:

(1) *The degree to which adolescents use social information* (as measured by the average degree of participants' adjustment of initial estimates towards social information).
(2) *Strategies deployed by adolescents when using social information* (as measured by participants' relative use of simpler all‐or‐nothing vs. compromising strategies to update initial estimates in response to social information).

In light of their reduced propensity for social affiliation (Blair et al., 2014; Viding & McCrory, 2019; Waller & Wagner, 2019) and their propensity to endorse social dominance (Pardini, 2011), we predicted that *adolescents with CP/HCU may demonstrate reduced social information use relative to CP/LCU and TD adolescents.* Given research showing that children and adolescents with CP are characterised by difficult peer relationships and demonstrate reduced social competence (Dodge et al., 1986; Ladd, 1990; Viding et al., 2009; Webster‐Stratton & Lindsay, 1999), and that those with CP/LCU may be particularly rigid in their social problem‐solving (Waschbusch et al., 2007), we predicted that *adolescents with CP, in particular those with CP/LCU, may be less likely to use compromising strategies than TD adolescents*.

## METHODS

### Participants

121 boys aged 11–16 were recruited from UK mainstream schools and specialised alternative provision (AP) schools for adolescents with behavioural difficulties. Screening questionnaires were administered to teachers enabling: a classification of current CP; dimensional assessment of CU traits; an overall screen for commonly co‐occurring symptoms with CP; and information regarding specialist education provision. Participants were presented with age‐appropriate information sheets and assent forms, which were also verbally explained. All parental consent/child assent procedures were in line with General Data Protection Regulation recommendations; the study was approved by the University College London Research Ethics Committee (Project ID number: 0622/001). Exclusion criteria included a diagnosis of autism spectrum disorder or presence of significant learning difficulties (a score of <70 on the Wechsler Abbreviated Scale of Intelligence, a measure of IQ (WASI; Wechsler, 1999)). Two CP participants were removed from descriptive analyses for failing to meet our inclusion criteria. Eight additional participants (five CP, three TD) were subsequently removed from main analyses due to task responding that was qualitatively different from expected task behaviour (see Section 2.2.3 for more detail). Thus final group Ns for descriptive analyses were: CP/HCU—34, CP/LCU—34, TD—48; final group Ns for main analyses were: CP/HCU—32; CP/LCU—31, TD—45.

### Measures

#### Participant characteristics

Participants with CP (*N* = 70) were required to meet age‐appropriate cut‐offs on the teacher‐version of the *Child and Adolescent Symptom Inventory* (CASI‐4R) Conduct Disorder Scale (Gadow & Sprafkin, 2005). The cut‐off scores associated with a clinical diagnosis of Conduct Disorder from teacher‐report according to the CASI manual are: a score of 3+ (ages 10–12), 4 + (ages 13–14), and 6+ (ages 15–16) on the CASI‐CD (Gadow & Sprafkin, 2005). Two CP participants were removed based on our exclusion criteria, leaving a CP group *N* of 68 for descriptive analyses (59 recruited from AP schools, 9 from mainstream schools).

CU traits were assessed using the *Inventory of Callous‐Unemotional Traits,* teacher‐version (ICU; Essau et al., 2006). Boys meeting CP criteria were further assigned to groups based on whether their ICU score was higher (CP/HCU; *N* = 34) or lower than/equal to the group median of 37 (CP/LCU; *N* = 34). We employed a median split approach to separate the children with CP to groups with high and lower levels of CU traits (HCU vs. LCU), for the following reasons:

1) Effects of CU traits do not often emerge as interactions and can instead lead to suppressor effects in correlational analyses (Frick, 2012).
2) The median split approach has, in the past, successfully delineated groups of children with CP who have different social‐cognitive processing patterns. The pattern of results in these two groups has often been such that, if they had been combined, researchers might have missed deficits in either group (Schwenck et al., 2012; Viding et al., 2012).
3) Suppressor effects can generate difficulties for interpretation, which mean that effects of CU traits may not emerge in interactions, although the CP/HCU and CP/LCU children look very different. The group centric analyses thus make it easier to interpret the translational relevance of findings, which is more challenging when examining suppressor effects in continuous analyses, for example, It is important to note that concerns regarding loss of power from dichotomizing relate to the case of bivariate normality (Cohen, 1983), but using continuous measure of CP and CU can generate problems if modelled together, given the absence of bivariate normality—high CU traits almost invariably denote high levels of CP, but not the other way around (Fontaine et al., 2011).

Based on prior published research, 37 represents a clinically meaningful cut‐off for HCU for both teacher and parent ratings (Docherty et al., 2017).

TD (*N* = 50 participants were recruited from mainstream schools and were required to score: (1) below the median score of the CP group on the ICU (which was 37), (2) within normal range (≤2) for the CASI, (3) within normal range (≥4) of the prosocial subscale of the Strengths and Difficulties Questionnaire (SDQ) and (4) below the cut‐off of 16 for teacher‐rated total difficulties (as per SDQ scoring norms; Youth in Mind 2016). Parent data for five TD participants on the following measures: CASI‐4R, ICU and SDQ, were included in lieu of missing teacher data (due to their being tested at home; see Section 2.2.2.). Two TD participants were removed due to incomplete data on the WASI (key for group matching), leaving a TD group *N* of 48 for descriptive analyses.

Data on age, IQ, and emotional and behavioural difficulties were collected from all participants individually during testing to ensure that these factors do not account for any significant differences between groups in our findings. We also included child‐rated measures of substance (alcohol and drug) use to ensure that these do not account for any findings, as substance use problems commonly co‐occur with CP (Wiesner et al., 2005). For more details about these measures and their scoring, and internal consistency in our sample, see Appendix S1.

Participants in the CP/LCU group were significantly younger than participants in the CP/HCU group (mean age 13.6 vs. 14.6). Main analyses were therefore carried out with and without age as a covariate. Participants were matched for IQ at a group level (see Table 1). For completeness, main analyses were also carried out with and without IQ as an additional covariate. See Appendix S2, Table S1, and Section 4.4 for more detail on covariate analyses. Table 1 summarises data on group matching and main participant characteristics. For full details of analyses see Appendix S3 and Table S2.

**TABLE 1:** Group matching and participant characteristics data

Characteristics and questionnaires	TD controls			CP/LCU			CP/HCU			*p* value	Post hoc^g^
	Mean (SD)	Min–max	*N*	Mean (SD)	Range	*N*	Mean (SD)	Min–max	*N*		
IQ (full score)^a^	90.4 (11.40)	70‐114	48	87.53 (10.30)	75‐119	34	84.85 (8.85)	72–107	34	.06	
Age (years)^b^	14.13 (1.26)	11.8–16.9	48	13.56 (1.38)	11.6–16.3	34	14.56 (1.22)	11.7–16.5	34	<.05**	2 < 3
CASI conduct disorder^b^	0.25 (0.64)	0‐2	48	6.55 (3.22)	3‐18	34	9.28 (4.76)	3–25	34	<.0001**	1 < 2 < 3
ICU^b^	19.31 (7.18)	2‐31	48	30.03 (5.73)	14‐37	34	46.68 (7.28)	38–63	34	<.0001**	1 < 2 < 3
Alcohol use and disorders^a^ ^,^ ^c^ ^,^ ^d^	47:1:0:0		48	33:0:1:0		34	29:4:1:0		34	.08	
Drug use and disorders^a^ ^,^ ^d^ ^,^ ^e^	47:1		48	30:4		34	28:6		34	.05** ^,^ ^f^	

Abbreviations: CASI, Child and Adolescent Symptom Inventory; CP/HCU, conduct problems and high levels of callous‐unemotional traits; CP/LCU, conduct problems and low levels of callous‐unemotional traits; ICU, Inventory of Callous And Unemotional traits; *N*, number of participants with complete measure; SD, standard deviation; SDQ*,* Strengths and Difficulties Questionnaire; TD, typically developing; WASI, Wechsler Abbreviated Scale of Intelligence. Where not stated, analyses were performed using one‐way ANOVA and post hoc tests were Bonferroni corrected for multiple comparisons. For summary of SDQ measures, see Table S2.

^a^ Measure obtained at testing phase, child report.

^b^ Measure obtained at screening phase, teacher report.

^c^ Counts for AUDIT risk categories (Low Risk:Increasing Risk:Higher Risk:Possible Dependence).

^d^ Assessed via Chi Square test.

^e^ Counts for DUDIT risk categories (Low Risk:Possible Drug Problems).

^f^ Significance at *p* = .05 did not remain after posthoc tests with bonferroni correction (see Appendix S1).

^g^ 1 = TD, 2 = LCU, 3 = HCU.

^**^ Results for comparisons smaller than or equal to this threshold.

#### Procedure

Participants were tested in a quiet room on their school premises or at home (5 TD participants). The experiment was programmed in *LIONESS Lab* (Giamattei et al., 2020), and presented on a Dell Latitude 7480 laptop. Experimental code is available on request. Data were collected as part of a larger battery of tasks.

#### The Berlin estimation AdjuStment task

**FIGURE 1:**
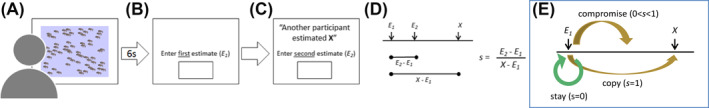
Task measuring social information use. Participants: (A) observe a group of animals for 6 seconds; (B) enter first estimate (*E*_1_) of total number of animals using computer keyboard; (C) observe social information (*X*): estimate of a (fictitious) participant from another school, alongside *E*_1_, and enter second estimate (*E*_2_); (D) Social information use per round (*s*) is calculated as degree of adjustment from *E*_1_ to *E*_2_, divided by distance between *E*_1_ and *X*; (E) Illustration of task strategies: stay (*s* = 0) and copy (*s* = 1) represent all‐or‐nothing strategies

The BEAST is a brief and simple perceptual judgement task, previously validated for use with adults (Molleman et al., 2019) and adolescents (Molleman et al., 2019). Figure 1 illustrates the task design (see Appendix S4 and Figure S1 for task instructions and example of one full trial). The task comprises five rounds in a fixed order. In each round, participants were presented with an image containing 43, 58, 34, 44 or 39 animals of different species for six seconds (importantly, the number of animals shown on each trial was chosen to ensure all five rounds were of a similar difficulty; Figure 1A). After the image had disappeared, participants were asked to make an initial estimate (*E*_1_) of the number of animals seen (Figure 1B). Importantly, the brief presentation time of images allowed an overall impression of the total number of animals, but prevented counting. No time limit was placed on entering *E*_1_. Following *E*_1*,*_ participants were presented with social information (*X*), and asked to provide a second estimate (*E*_2_, Figure 1C). Social information use was characterised as degree of adjustment towards *X*, that is participants' adjustment of *E*_1_ when making *E*_2_ (Figure 1D). For each round, the relative distance (*s*) that a participant moved towards *X* was calculated as *s *= (*E*_2_ − *E*_1_)/(*X* − *E*_1_). Reordered as *E*_2_ = (1 − *s*) ⋅ *E*_1_ + *s* ⋅ *X*, this shows that *E*_2_ in each round is an average of *E*_1_ and *X*, weighted by *s*. Adjustments were classified as compromising if participants' *E*_2_ fell between their *E*_1_ and *X*, and all‐or‐nothing if they stuck with *E*_1_ when making *E*_2_, or directly copied *X* (Figure 1E).

Participants were informed that the social information seen on each round was the estimate of an adolescent participant at another school. In reality, *X* was calibrated to participants' *E*_1_ in a way that allowed for a relatively constant scope for adjustment in each round while experimentally controlling for possible ‘distance weighting’ effects, the observation that people tend to discount information deviating too strongly from initial estimates (Moussaïd et al., 2013; Appendix S5). This minor deception was approved by the UCL ethics committee (project code: 0622/001).

Participants were informed that they would earn points based on their accuracy. To ensure that participants could not learn their own skill or the accuracy of the social information provided across the five task rounds, no feedback was given to participants about their performance. This enabled as unbiased an estimate of social information use as possible (Molleman et al., 2019). Participants were not rewarded for their participation.

Following Molleman et al. (2019), prior to calculating *s*, we excluded data from all rounds where a participant made adjustments considered qualitatively different from expected task behaviour: giving negative weight to *X* (*s* < 0; 23 cases 3.8% of all data]), or not determining *E*_2_ as a weighted average of *E*_1_ and *X* (*s* > 1; 50 cases [8.4% of data]). Data from eight adolescents (two CP/HCU, three CP/LCU and three TD) were excluded from the main analyses for giving three or more responses out of the five task rounds that met these criteria. Following these data cleaning procedures, final group Ns for the main analyses were: CP/HCU—32; CP/LCU—31, TD—45.

## STATISTICAL ANALYSES

For full details of statistical analyses of demographic and experimental data, please refer to Appendix S6, Table S2, and Tables 1 and 2.

**TABLE 2:** Model 1—linear mixed effects model fitted to participants' mean adjustment (*S*) across five rounds by group with subject as a random factor. Model 2—generalized linear mixed model fitted to decisions to use an all‐or‐nothing strategy (copy/stay) (coded as 1) or a compromising strategy (weighted combination of initial estimate and social information) (coded as 0) by group with subject (ID) as a random factor

Predictors	Model 1			Model 2		
	Estimates	CI	*p*	Odds ratios	CI	*p*
(Intercept)	0.36	.28–.43	<.001***	0.42	.23–.91	.013*
Group (CP/LCU)	−0.04	−.16–.08	.500	5.78	1.77–15.98	.002**
Group (CP/HCU)	0.00	−.12–.12	.963	2.20	.69–6.06	.152
Random effects						
*σ* ^2^	0.06			3.29		
*τ*_00_	0.06_ID_			3.96_ID_		
ICC	0.48			0.55		
*N*	108_ID_			108_ID_		
Observations	499			499		
Marginal *R* ^2^/conditional *R* ^2^	0.003/0.481			0.068/0.577		

Abbreviations: CP/HCU, conduct problems and high levels of callous‐unemotional traits; CP/LCU, conduct problems and low levels of callous‐unemotional traits; ICC, intraclass‐correlation; *N*, number of participants; *σ* ^2^, residual variance, *τ*_00_, random slope (between‐group) variance.

***p < .0001, **p <.001, *p < .05.

## RESULTS

### Basic behavioural results

Participants' initial estimate tended to be lower than the true value (averages as percentage of true value: CP/HCU (74%), CP/LCU (68%), TD (69%); see Figure S2, panel A), a common observation in similar tasks (Molleman et al., 2019, 2020). Groups did not differ in the accuracy of initial estimates (see Figure S2; *F*(2, 105) *=* 0.88*, p* = .42; *η* ^2^ = 0.02).

### Social information use

**FIGURE 2:**
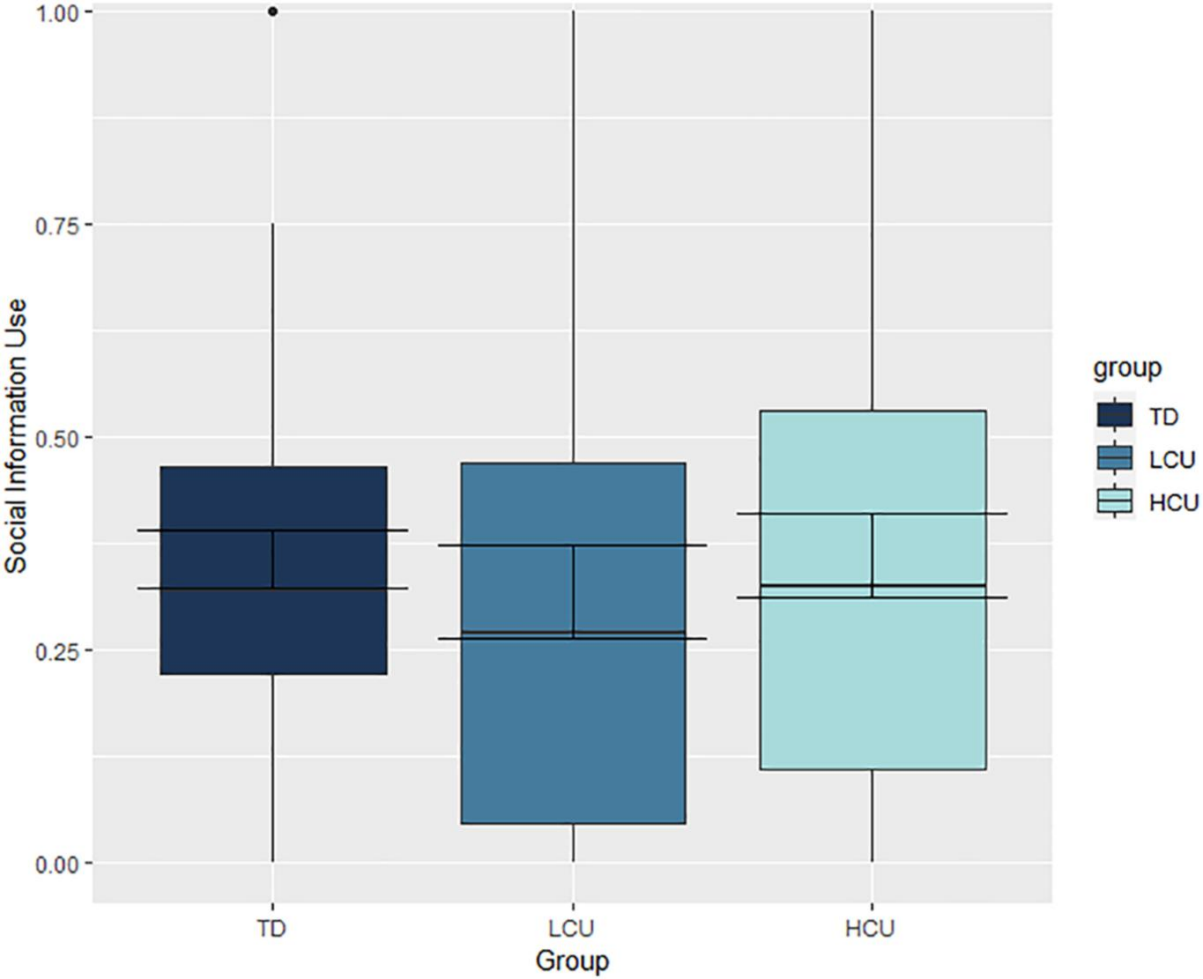
Social information use by group. Error bars represent standard‐error. Box boundaries represent the first and third quartiles of data. Whiskers represent 1.5 × the interquartile range. CP/HCU, conduct problems and high levels of callous‐unemotional traits; CP/LCU, conduct problems and low levels of callous‐unemotional traits; TD, typically developing

In contrast with predictions, groups did not significantly differ on how much they adjusted their estimates following social information (*p* = .75; further details in Table 2 (Model 1), and Figure 2; model specification and assumption checks in Appendix S7, Figure S3. For covariate analysis with age and IQ, see Appendix S2 and Table S1. Mean adjustments across trials were less than 0.5 across groups (CP/HCU—0.36 (SD = 0.28), CP/LCU—0.32 (0.30), TD—0.36 (0.23)) implying that participants assigned more weight to their own views than to the social information (Figure 2). This is also reflected on a trial level (proportion of trials *s* < 0.5: CP/HCU—68.75%, CP/LCU—74.19%, TD—80.00%). The majority (92.41%) of participants' data fell within 0 ≤ s ≤ 1 (Figure S4), and within‐participant variation in adjustments was smaller than between‐participant variation (Table S3).

### Strategy use

**FIGURE 3:**
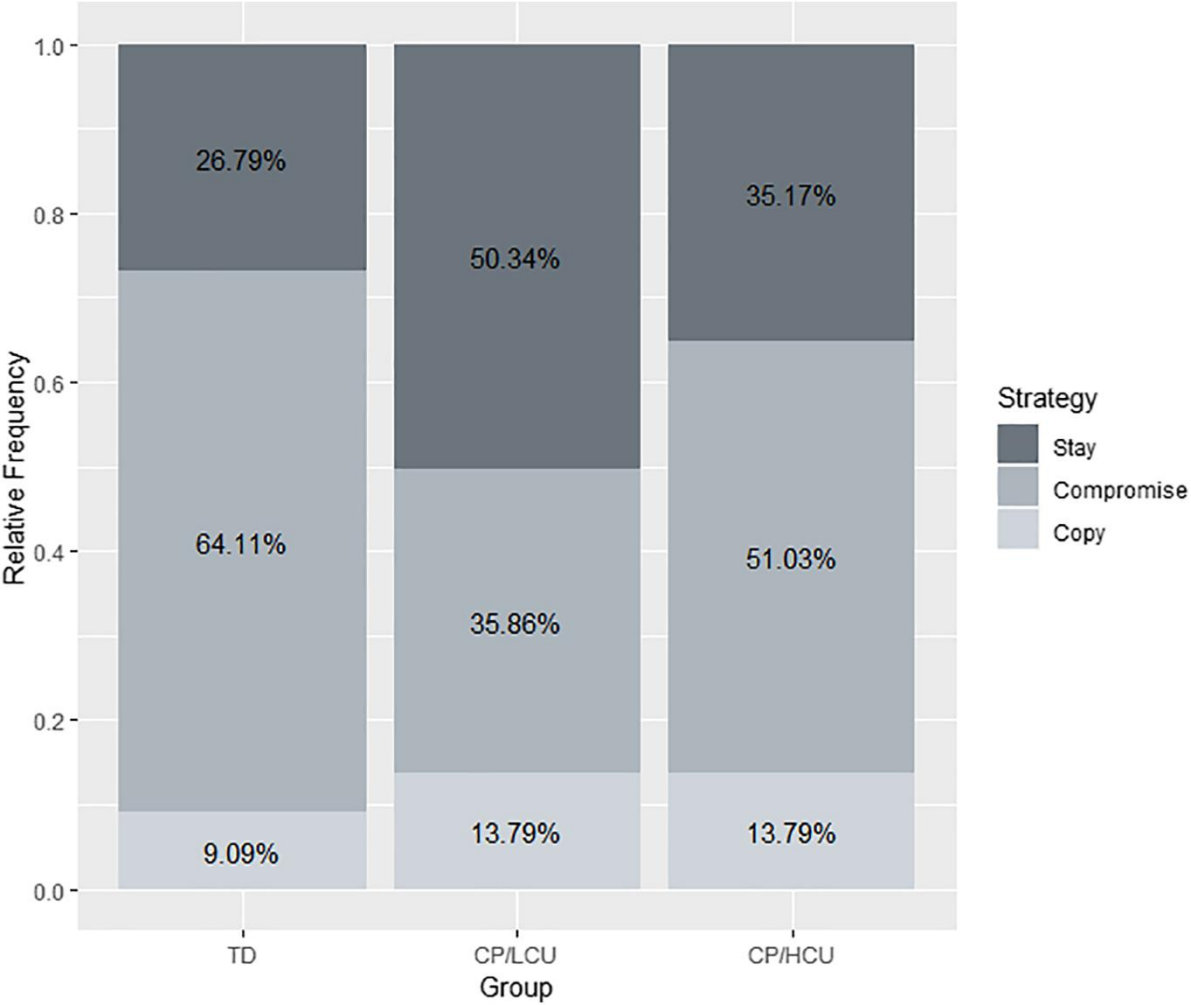
Relative frequency of strategy use by group. TD, typically developing; CP/LCU, conduct problems and low levels of callous‐unemotional traits; CP/HCU, conduct problems and high levels of callous‐unemotional traits

In line with predictions, a significant group difference in relative use of all‐or‐nothing versus compromising strategies was observed ($\chi^{2}$ = 9.93, *p* = .007; further details in Table 2 (Model 2) and Figure 3; model specification and assumption checks in Appendix S7 and Figure S3). Post‐hoc Tukey comparisons revealed that this was driven by the CP/LCU group using a significantly lower proportion of compromising strategies than the TD group (*p* = .005). The CP/LCU group chose compromising strategies 35.86% of the time compared to 64.11% in the TD group (see Figure 3), being more likely either to stick with their original estimate or copy the social information. There was no statistically significant difference in strategy use between CP/LCU and CP/HCU (*p* = .24), or CP/HCU and TD (*p* = .43) groups. To ensure that the observed group difference cannot be accounted for by the age difference between CP/LCU and CP/HCU groups, nor by group IQ, the model was re‐run with age and IQ included as covariates. The main effect of group remained significant ($\chi^{2}$  = 8.72, *p* = .013), and was still driven by a difference between CP/LCU and TD groups (*p* = .01; model and full results in Appendix S2 and Table S1). Inspection of the frequency of different strategy usage by group (Figure 3) led us to run exploratory analyses to investigate whether groups differed in their number of ‘stay’ responses. A significant group difference in stay (vs. copy and compromising) responses was observed (*χ* ^2^ = 7.53, *p* = .023), driven by the CP/LCU group choosing ‘stay’ responses more frequently than the TD group (*p* = .02). No group difference in ‘copy’ (vs. stay and compromising) responses was observed (*χ* ^2^ = 0.21, *p* = .90).

We ran three further models to examine whether variations in cognitive empathy, affective empathy, and cognitive perspective taking might account for findings. This led to no change in results (see Appendix S8, Tables S4 and S5 for summary of measures and full models). Additionally, we used Spearman's Rho correlation analysis to examine how group membership, substance use, and SDQ rated emotional problems, peer problems, hyperactivity, and total difficulties related to strategy use. No statistically significant association was observed between strategy use and these measures. (Table S6), so no further covariate analyses were run.

## DISCUSSION

Using a brief and simple estimation updating task, we assessed two important aspects of social information use in adolescents with CP/HCU, CP/LCU, and TD peers: *degree* of social information use, and *strategy* when using social information. We hypothesised that: (1) adolescents with CP/HCU would use social information to a lesser degree than other groups and (2) adolescents with CP, in particular those with CP/LCU, would be less likely to adopt compromising strategies when using social information. We found no support for the first hypothesis: there were no group differences in degree of social information use. However, in line with our second hypothesis, a group difference was observed in strategy adopted when using social information. CP/LCU participants were less likely to use compromising strategies relative to TD participants.

Our prediction that adolescents with CP/HCU would use social information to a lesser degree than other groups was based on research demonstrating a lower propensity for social affiliation (O’Nions et al., 2017; Sakai et al., 2016) and a higher likelihood of endorsing dominant social goals in CP/HCU adolescents relative to CP/LCU (Pardini, 2011). We therefore reasoned that they may be less likely to incorporate social information into their behaviour. However, two key aspects of our study may explain why we did not find the predicted pattern of performance in the CP/HCU group. First, studies that have demonstrated atypical processing of affiliative signals in CP/HCU group have used affective stimuli (Hodsoll et al., 2014; O'Nions et al., 2017), whereas the BEAST task did not require processing of affect. Second, the study that demonstrated endorsement of dominant social goals looked at hypothetical conflict situations using vignettes (Pardini, 2011), whereas our measure of social information use was more abstract—feedback from an anonymous other. Our findings thus suggest that in the absence of affect or potential conflict, and when provided time to deliberate, CP/HCU adolescents do not differ from their peers in social information use. Future studies could explore whether this is also the case when CP/HCU adolescents are making affective judgments (e.g. judging the emotion of a face and seeing another person's judgment), or whether manipulating the source of the social information might impact task behaviour in this group (e.g. providing information from ‘a person who is very good at similar tasks’ to introduce a competitive element to the task).

Although *degree* of adjustment in response to social information was similar across groups, our analyses revealed that *strategies* used to adjust estimates differed. Specifically, adolescents with CP/LCU compromised significantly less than TD adolescents when incorporating social information into their initial judgments. Interestingly, this appears to be driven by this group sticking with their initial responses as opposed to copying the social information. This complements the finding of Waschbusch et al. (2007) that CP/LCU is associated with poorer and more rigid social problem‐solving. We propose that less compromising responses to others' feedback might generate difficulties in social interactions for these adolescents. For example, an unwillingness to accept or find middle‐ground with another person's point of view could be perceived as hostile, a trait strongly associated with aggression (Buss & Perry, 1992). Future work could build on this by examining social information use in more ecologically valid contexts (e.g. using information from a known other or affectively charged information). Further research might also help elucidate whether the reduced compromising observed in the CP/LCU group in this study, as well as the propensity for rigid responding demonstrated by children with CP and lower levels of CU traits in the study of Waschbusch et al. (2007), relates to social information specifically, or might reflect a general lack of flexibility. One way to explore this would be to look at the relationship between performance on this task and executive functioning, as relationships have been demonstrated between both CP (Ogilvie et al., 2011) and CU traits (Platje et al., 2018) and deficits in this domain in adolescence. CP/LCU has been suggested to be linked with experience of hostile and inconsistent parenting and to be associated with increased stress reactivity, threat and frustration‐triggered aggression, and difficulties in emotion regulation (Blair et al., 2014; Frick et al., 2014; Lovallo, 2013). It is plausible that early life experiences of adolescents with CP/LCU could also contribute to difficulties in optimally integrating information from others, perhaps partly due to difficulties in trusting others. The current study, along with the study of Waschbusch et al. (2007) clearly highlights the need for more research directly investigating social‐cognitive processing in CP/LCU relative to CP/HCU and TD peers.

It is important to note some limitations of the current study. First, this study focused only on males. We chose to do this because CP is more prevalent in males, and there are studies suggesting that aetiology of both CP and CU may differ for males and females (Fontaine et al., 2010). Future work should also examine social information use in females with CP. Second, it may be helpful to include both parent and teacher ratings of CP and CU in future studies, as opposed a single rater. Third, our sample size was constrained by the difficulty of working with a hard to reach population that are challenging to recruit/engage in research (young people with CP). Although this sample size is typical of studies in the field (Hodsoll et al., 2014; Schwenck et al., 2012), it is important to bear this in mind when considering our results, and we would like to highlight the need for replication of this study before strong conclusions are drawn based on these findings. Fourth, we would like to acknowledge the lack of agreed upon cut‐off criteria for use of the ICU measure (assessing CU traits). This may limit comparison across studies that use a person‐centred approach. Additionally, future work could expand the range of domains and measures assessed, as well as exploring how different sources and types of social information impact performance on the BEAST task. In relation to measures, future studies might benefit from assessing executive functioning (as discussed above). It may also be of interest to include diary assessments of aggression and prosocial behaviour/friendship measures to probe how the task relates to social functioning, as well as measures of suggestibility to give more insight into factors that may be driving task responding. In relation to the source and type of social information received by participants, as noted above, future studies could contrast use of different kinds of social information for example affectively charged versus non‐affectively charged information. Including information from a human confederate source might also better mimic real‐world contextual cues that may influence adolescent decision making. Finally, it is worth noting that the way that the social information was framed in the current study (as coming from ‘another child at another school’) may have created school‐based allegiances whereby participants viewed the ‘other’ as belonging to an out‐group; this is known to be important in adolescent decision making (for example Horn [2006]), and to affect performance on social information processing tasks with adults (for example Izuma and Adolphs 2013]). It may therefore be worthwhile to investigate whether social information from in‐group (e.g. same school) and out‐group (e.g. other school) sources impacts task performance in these groups.

## CONCLUSION

To our knowledge, this is the first study to investigate social information use in adolescents with CP. Although overall degree of social information use was similar across groups under the conditions of our study, adolescents with CP/LCU used fewer compromising strategies than TD adolescents when integrating this information with their initial beliefs. This main finding indicates that practice considering ‘middle grounds’ between their thoughts and those of others might be a potential target for behaviour management for adolescents with CP/LCU. However, more research is needed in order to establish this, including replication of this study, research with adolescent girls with CP, and research using different forms of social information. Overall, our observations add to what we already know about social information processing in CP and also motivate future research so that we can develop a more nuanced understanding of the social‐cognitive differences between adolescents with CP and their peers. This study further highlights the importance of acknowledging and investigating heterogeneity among adolescents with CP. A more comprehensive understanding of both commonalities and differences among different adolescents with CP, as well as their profile of social‐cognitive strengths and weaknesses, has the potential to inform tailored clinical interventions and behaviour management practices.

## CONFLICT OF INTEREST

Essi Viding is a member of the Editorial Advisory Board for JCPP *Advances*. The remaining authors have declared that they have no competing or potential conflicts of interest. [Corrections made on 22 June 2022, after first online publication: This Conflict of Interest statement has been updated in this version.]

## AUTHOR CONTRIBUTIONS

**Anne Gaule:** Investigation; data curation; formal analysis; visualization; Writing – original draft; Writing – review & editing. **L. Bevilacqua:** Investigation; project administration; data curation; Writing review & editing. **L. Molleman:** Conceptualisation; funding acquisition; formal analysis; visualization methodology; software; Writing – review & editing. **R. Roberts:** Investigation; project administration; data curation; Writing – review & editing. **A. C. van Duijvenvoorde:** Conceptualisation; funding acquisition; Writing – review & editing. **W. van den Bos:** Conceptualisation; funding acquisition; methodology; software; Writing – review & editing. **E. J. McCrory:** Conceptualisation; Writing – original draft; Writing – review & editing. **E. Viding:** Conceptualisation; supervision; project administration; Writing – original draft; Writing – review & editing.

## ETHICAL CONSIDERATION

This study was performed in line with the principles of the Declaration of Helsinki. Approval was granted by the University College London Research Ethics Committee (0622/001).

## Supporting information

Supporting Information S1

## Data Availability

The data that support the findings of this study are available on request from the corresponding author. The data are not publicly available due to privacy or ethical restrictions. All experimental code available upon request.
