# Supplementary material for: Social information use in adolescents with conduct problems and varying levels of callous‐unemotional traits
Source: JCPP Adv. 2022 Mar 5;2(1):e12067. doi: 10.1002/jcv2.12067 (PMC10242950; doi:10.1002/jcv2.12067)
Supplement: Supplementary file 1 — Supporting Information S1 [file JCV2-2-e12067-s001.docx]

­­Social Information Use in Children with Conduct Problems and Varying Levels of Callous Unemotional Traits: Supplemental Information

## FigS1 Example of one full experimental trial


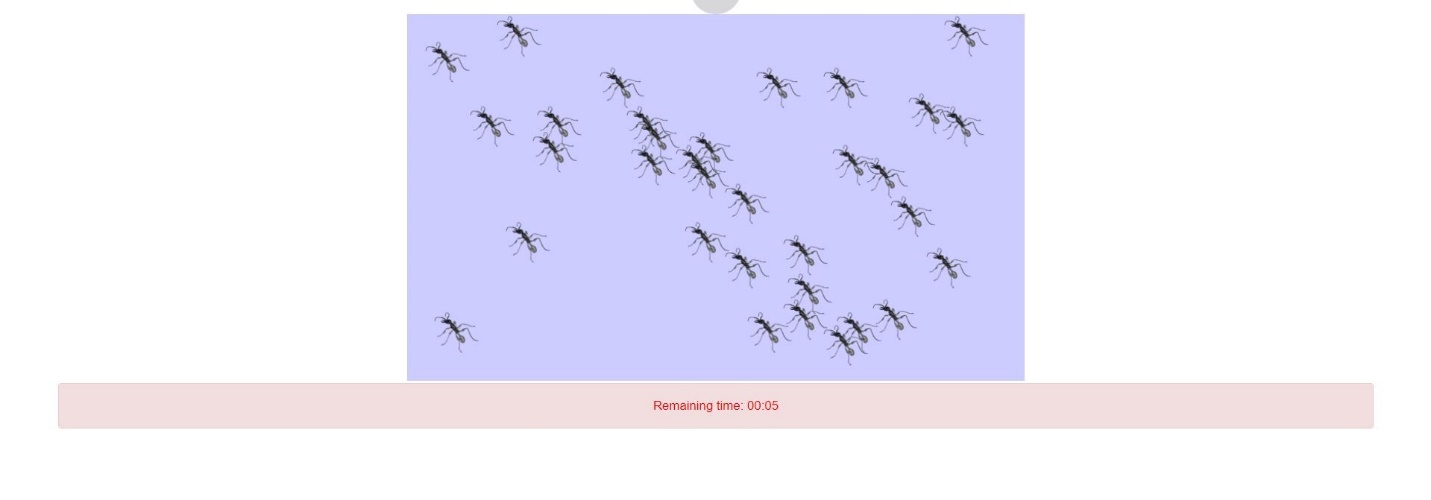
**One experimental round:**

**BEAST task running.** Round 1 screen 1 (zoomed in for clarity)


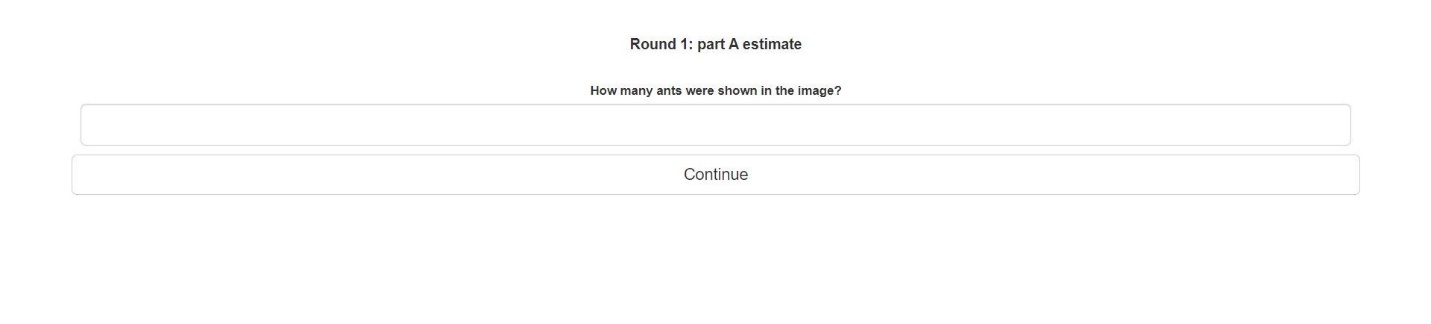


**BEAST task running.** Round 1 screen 2 (zoomed in for clarity)


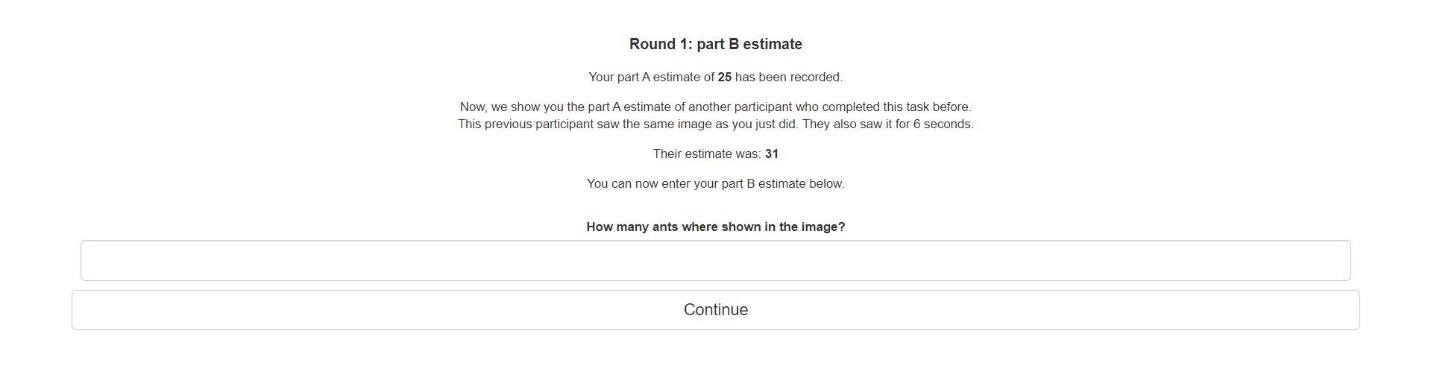


**BEAST task running.** Round 1 screen 3 (zoomed in for clarity)

## FigS2 Accuracy in First and Second Estimates By Group

**A**


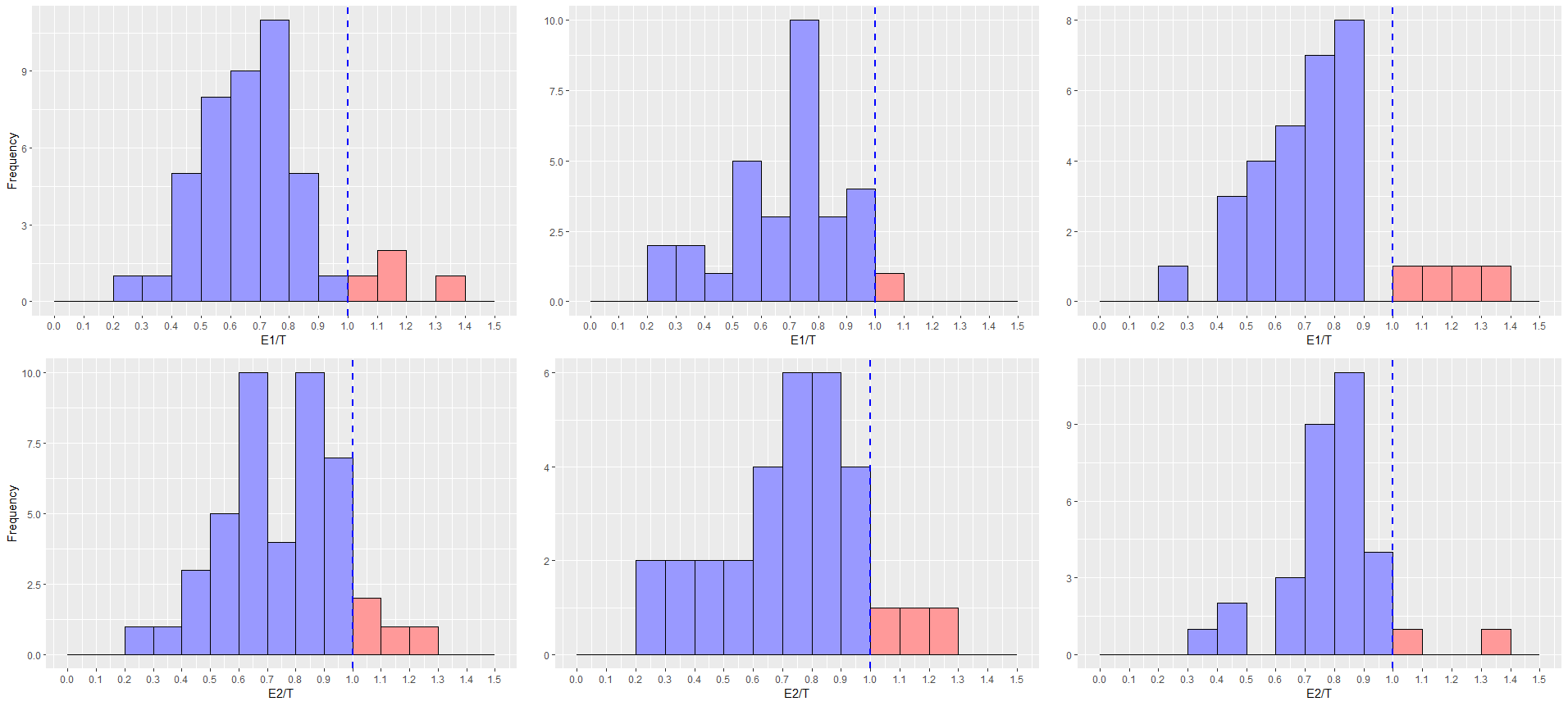


**B**

**Accuracy in first and second estimates by group.** Panels show frequency distributions of participants’ estimates before (E1; panel A) and after (E2; panel B) receiving social information for each group (TD, CP/LCU, CP/HCU). Values were standardised by dividing E1 over the true value for each of the trials (which varied between 30 and 60 animals). The mean first estimates were all underestimates of the true values and were as follows: TD group - 69%, CP/LCU group – 68%, CP/HCU group – 74%. Accuracy of initial estimates between groups was compared using a one-way ANOVA – no group difference in accuracy was observed (*F*(2,105) = 0.88, *p* = 0.42, η^2^ = 0.02).

##
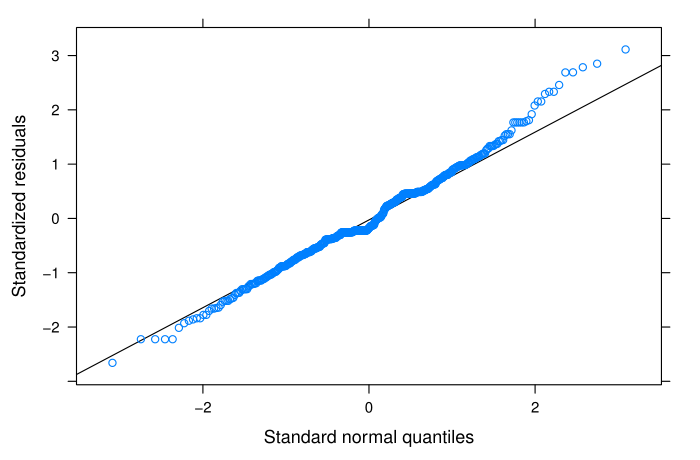
FigS3 Model Assumption Checks

A.

B.

**
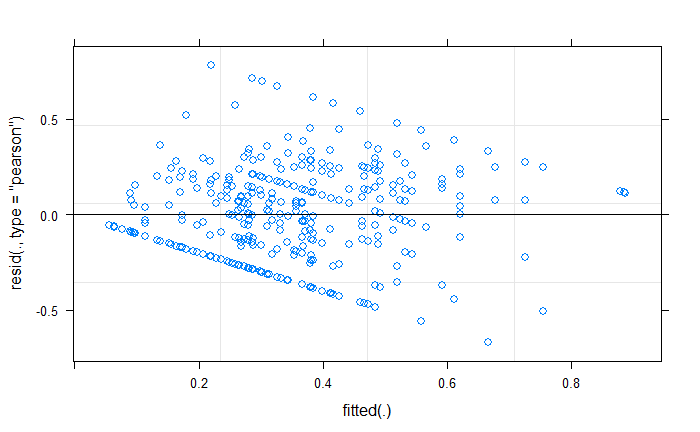
**

**
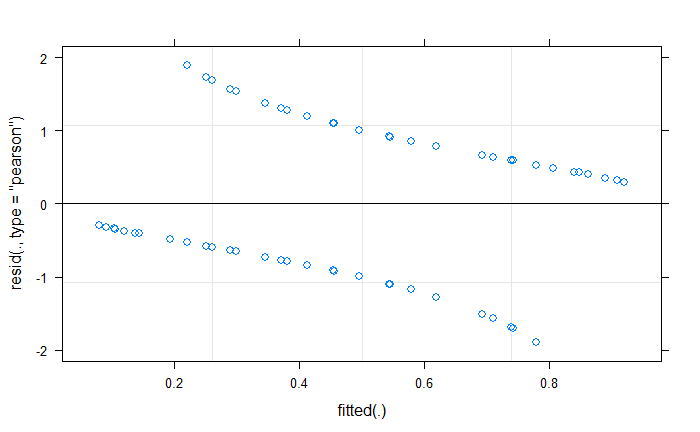

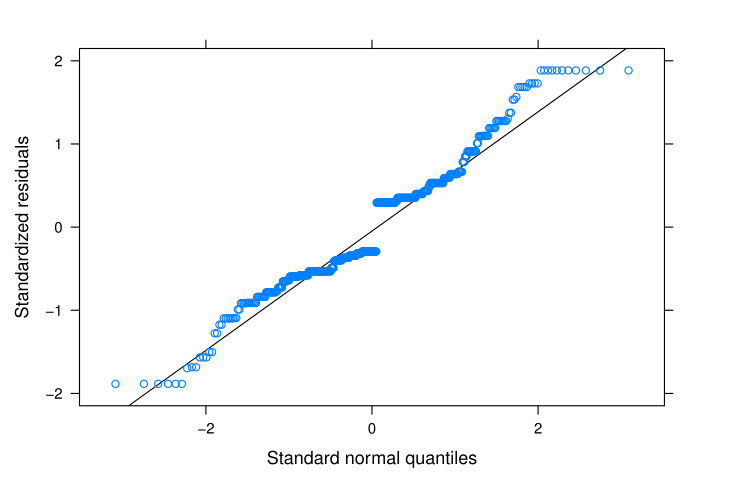
**

C.

D.

**Residual plots for Models 1 & 2.** Plots **A.** and **B.** are scatter and qq plots respectively, demonstrating that model 1 residuals are fairly, although not perfectly, normally distributed. Linear Mixed Effects models have been demonstrated to be robust against deviations in distributional assumptions (e.g. Schielzeth et al. 2020), we therefore consider this model appropriate for our data. A Levene test revealed no homoscadicity of residuals (*F*(2, 496) = 0.07, *p =* 0.94). Plots **C.** and **D.** are scatter and qq plots respectively, demonstrating that model 2 residuals are fairly, although not perfectly, normally distributed. Generalised Linear Mixed Models have been demonstrated to be robust against deviations in distributional assumptions (Schielzeth et al., 2020). A levene test revealed no homoscadicity of residuals (*F*(2, 496) = 1.04, *p =* 0.35).

## FigS4 Social Information Use - Adjustments per Round (All Cases Included)

**
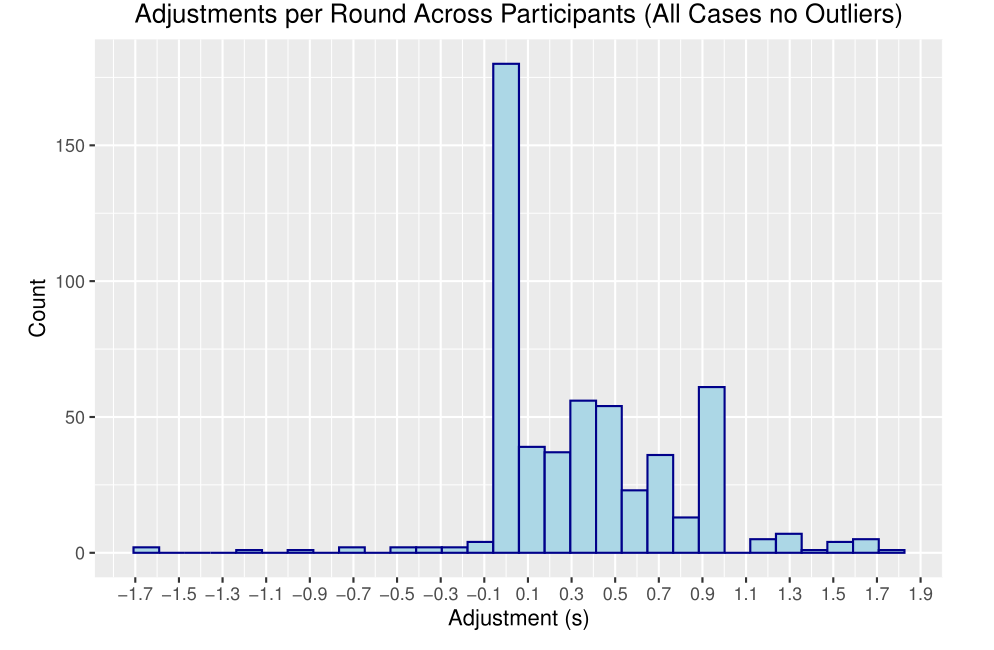
**

**Histogram showing frequency of adjustments per round including qualitatively different responding i.e. outside range of 0 ≤ s ≤ 1 (removed for main analyses).** Two data-points were removed when preparing this graph for being more than three standard deviations from the mean (outliers).

## Table S1 Age and IQ Covariate Analysis - Model Results:

|  | *Chi Squared* | *Df* | *p-value* |
| --- | --- | --- | --- |
| ***Model 1*** |  |  |  |
| *Group* | *0.96* | *2* | *0.62* |
| *IQ* | ***3.90*** | ***1*** | ***0.048**** |
| *Age* | *0.24* | *1* | *0.63* |
|  |  |  |  |
| ***Model 2*** |  |  |  |
| *Group* | ***8.72*** | ***2*** | ***0.013**** |
| *IQ (Z score)* | *0.995* | *1* | *0.32* |
| *Age (Z score)* | *0.00* | *2* | *0.998* |
|  |  |  |  |

***Table 4b. Analysis of Deviance (Type II Wald) tests of main models with age and IQ as covariates****. Model 1 is a linear mixed model fitted to participants' mean adjustment (S) across the five rounds by group (Conduct problems (CP) with High callous-unemotional (CU) traits, CP with low CU traits, typically developing) with age and IQ included as covariates. Model 2 is a logistic generalized mixed model (GLMM) fitted to decisions to use an extreme heuristic (copy/stay) (coded as 1) or a compromising heuristic (coded as 0) with age and IQ included as covariates. Age and IQ scores were normalized to improve model fit.*

## Table S2 Additional Participant Characteristic Data – Strengths and Difficulties

| **Strengths and Difficulties** ^a^ | **TD controls** | | | **CP/LCU** | |  | **CP/HCU** | | | **P value** | **Post hoc*** |
| --- | --- | --- | --- | --- | --- | --- | --- | --- | --- | --- | --- |
|  | **Mean (SD)** | **Min-Max** | **N** | **Mean (SD)** | **Min-Max** | **N** | **Mean (SD)** | **Min-Max** | **N** |  |  |
|  |  |  |  |  |  |  |  |  |  |  |  |
| Hyper-activity ^b^ | 2.31 (2.01) | 0-6 | 48 | 6.50 (2.73) | 1-10 | 34 | 7.92 (2.31) | 2-10 | 33 | <.05** | 1 < 2 < 3 |
|  |  |  |  |  |  |  |  |  |  |  |  |
| Emotional difficulties ^b^ | 1.00 (1.56) | 0-5 | 48 | 3.53 (2.94) | 0-10 | 34 | 3.88 (2.81) | 0-10 | 34 | <.0001** | 1<2, 1<3 |
|  |  |  |  |  |  |  |  |  |  |  |  |
| Peer problems^b^ | 1.11 (1.48) | 0-5 | 48 | 2.91 (1.96) | 0-8 | 34 | 3.54 (2.53) | 0-9 | 34 | <.0001** | 1<2, 1<3 |
| Prosocial behaviour | 7.75 (2.08) | 4-10 | 48 | 5.70 (2.44) | 0-10 | 34 | 2.87 (1.53) | 0-5 | 34 | <.0001** | 3 < 2 < 1 |
|  |  |  |  |  |  |  |  |  |  |  |  |
| Total difficulties | 4.85 (3.68) | 0-15 | 48 | 17.26 (6.64) | 3-32 | 34 | 21.44 (5.80) | 7-35 | 33 | <.01** | 1 < 2 < 3 |

Table summarising strength and difficulties scores including subscales and total difficulties. TD typically developing, CP/LCU conduct problems & low levels of callous unemotional traits, CP/HCU conduct problems and high levels of callous unemotional traits, SD standard deviation, N number of participants with complete measure. Where not stated, analyses were performed using one-way ANOVA and post hoc tests were Bonferroni corrected for multiple comparisons.

’*’ 1 = TD, 2 = LCU, 3 = HCU.

** Results for comparisons smaller than or equal to this threshold

^a^ All measures obtained at screening phase, teacher report.

^b^ Assessed via three pairwise Mann-Whitney U tests due to violation of ANOVA assumptions. Directionality inferred through visual inspection of means.

## Table S3 Within vs Between Participant Variation In Adjustments (*s*)

| **Group** | **Between Ppt Standard Deviation in *s*** | **Within Ppt Standard Deviation in *s*** |
| --- | --- | --- |
| TD | 0.31 | 0.21 |
| LCU | 0.38 | 0.20 |
| HCU | 0.36 | 0.20 |

**Standard deviation in adjustments (*s*) between participants in each group and within participants in each group.** TD - typically developing, CP/LCU conduct problems and low levels of callous-unemotional traits, CP/HCU conduct problems and high levels of callous-unemotional traits.

## TableS4 Additional Covariate Measures – Descriptive statistics

| **Supplemental Measures^a^** | **TD controls** |  |  | **CP/LCU** |  |  | **CP/HCU** |  |  | **P value** | **Post hoc*** |
| --- | --- | --- | --- | --- | --- | --- | --- | --- | --- | --- | --- |
|  | **Mean (SD)** | **Min-Max** | **N** | **Mean (SD)** | **Range** | **N** | **Mean (SD)** | **Min-Max** | **N** |  |  |
|  |  |  |  |  |  |  |  |  |  |  |  |
| **Cognitive Perspective Taking** | 17.08 (4.16) | 9-26 | 45 | 15.88 (5.78) | 7-28 | 32 | 13.69 (2.31) | 2-21 | 33 | 0.01* | 1 > 3 |
|  |  |  |  |  |  |  |  |  |  |  |  |
| **Cognitive Empathy** | 35.02 (4.85) | 22-45 | 46 | 32 (4.36) | 22-44 | 28 | 34.35 (3.55) | 28-42 | 26 | 0.02* | 1 > 2 |
|  |  |  |  |  |  |  |  |  |  |  |  |
| **Affective Empathy** | 33.99 (5.84) | 22-45 | 46 | 32.51 (6.43) | 22-44 | 28 | 32 (6.39) | 20-49 | 26 | 0.37 |  |

Table summarising scores on extra measures included as covariates.TD typically developing, CP/LCU conduct problems & low levels of callous unemotional traits, CP/HCU conduct problems and high levels of callous unemotional traits, SD standard deviation, N = number of participants that completed measure. Analyses were performed using one-way ANOVA and post hoc tests were Bonferroni corrected for multiple comparisons.

’*’ 1 = TD, 2 = LCU, 3 = HCU.
** Results for comparisons smaller than or equal to this threshold
^a^ All measures obtained at testing phase, child report

## Table S5 Additional Covariate Model Results

|  | *Chi Squared* | *Df* | *p-value* |
| --- | --- | --- | --- |
| ***Model 3 – Social information use*** |  |  |  |
| *Group* | *0.07* | *2* | *0.97* |
| *BES Cognitive Empathy* | *0.65* | *1* | *0.42* |
|  |  |  |  |
| ***Model 4 – Strategy Use*** |  |  |  |
| *Group* | ***7.13*** | ***2*** | ***0.03**** |
| *BES Cognitive Empathy* | *0.00* | *2* | *0.997* |
| ***Model 5 – Social information use*** |  |  |  |
| *Group* | *0.26* | *2* | *0.88* |
| *BES Affective Empathy* | *0.01* | *1* | *0.94* |
|  |  |  |  |
| ***Model 6 – Strategy Use*** |  |  |  |
| *Group* | ***7.75*** | ***2*** | ***0.02**** |
| *Affective Empathy* | *0.04* | *2* | *0.84* |
| ***Model 7 – Social information use*** |  |  |  |
| *Group* | *0.36* | *2* | *0.83* |
| *Cognitive Perspective Taking* | *0.27* | *1* | *0.60* |
|  |  |  |  |
| ***Model 8 –Strategy Use*** |  |  |  |
| *Group* | ***9.45*** | ***2*** | ***0.01**** |
| *Cognitive Perspective Taking* | *0.16* | *2* | *0.69* |

Table of Analysis of Deviance (Type II Wald) tests of main models with additional variables included as covariates. *Model 1* is a linear mixed model fitted to participants' mean adjustment (S) across the five rounds by group (Conduct problems (CP) with High callous-unemotional (CU) traits, CP with low CU traits, typically developing) with cognitive empathy included as a covariate. *Model 2* is a logistic generalized mixed model (GLMM) fitted to decisions to use an extreme heuristic (copy/stay) (coded as 1) or a compromising heuristic (coded as 0) by group with cognitive empathy as a covariate. *Model 3* is a linear mixed model fitted to S across 5 rounds by group, with affective empathy included as a covariate. *Model 4* is a GLMM fitted to decisions to use an extreme heuristic by group with affective empathy taking included as a covariate. *Model 5* is a linear mixed model fitted to S across 5 rounds by group, with cognitive perspective taking included as a covariate. *Model 6* is a GLMM fitted to decisions to use an extreme heuristic by group with cognitive perspective taking included as a covariate.

## Table S6 Additional covariate analyses

| \|  \| **Kendall’s Tau** \| \| **P Value** \| **Adjusted P Value** \| \| --- \| --- \| --- \| --- \| --- \| \| Extreme Responding \| 1.00 \| 0.00 \| \| 0.00 \| \| Child Group \| 0.14 \| 0.07 \| \| 0.19 \| \| SDQ Emotional Problems \| **0.18** \| **0.02*** \| \| **0.08** \| \| SDQ Peer Problems \| 0.12 \| 0.12 \| \| 0.24 \| \| SDQ Hyperactivity \| 0.02 \| 0.80 \| \| 0.84 \| \| SDQ Total Difficulties \| 0.10 \| 0.17 \| \| 0.27 \| \| AUDIT (alcohol use) \| -0.03 \| 0.74 \| \| 0.84 \| \| DUDIT (drug use) \| -0.02 \| 0.84 \| \| 0.88 \| |  |  |
| --- | --- | --- | --- | --- | --- | --- | --- | --- | --- | --- | --- | --- | --- | --- | --- | --- | --- | --- | --- | --- | --- | --- | --- | --- | --- | --- | --- | --- | --- | --- | --- | --- | --- | --- | --- | --- | --- | --- | --- | --- | --- | --- | --- | --- | --- | --- | --- |

**Kendall's Tau-B correlations between proportion of all-or-nothing responding relative to compromising responding and measures of commonly co-occurring symptoms with CP.** *p* values were corrected for multiple comparisons using the Benjamin-Hochberg correction. The initial modest correlation between all-or-nothing responding and emotional problems (*τ* = 0.18, *p* = 0.02) revealed by Kendell’s Tau-B analyses did not survive the correction for multiple comparisons.

## Appendix S1 Supplemental Methods – Measure details and internal consistency

Conduct problems were assessed using the *Child and Adolescent Symptom Inventory* (CASI-4R; Gadow & Sprafkin 2005) Conduct Disorder Scale (CASI-CD). This scale contains nine items rated on a 4-point scale from 'Never' to 'Very often'. The measure showed good internal consistency in our sample ($\alpha$ = 0.89).

Callous and Unemotional Traits were assessed using all items from the *Inventory of Callous-Unemotional Traits* (ICU; Essau, Sasagawa, & Frick 2006). The ICU contains 24 items rated on a 4-point scale from 'not at all true' to 'definitely true'. The total *sum* score was used to identify CU groups. The measure showed very good internal consistency in our sample ($\alpha$= 0.94).

IQ was assessed using the two-subtest version of the WASI (Wechsler, 1999). Substance use was assessed via the Alcohol Use Disorders Identification Test (AUDIT; Babor et al. 2001) and the Drug Use Disorders Identification Test (DUDIT; (Berman et al., 2005b). The AUDIT and DUDIT include 10 and 11 items respectively, and measure substance use, harmful use, and symptoms of dependence. The first items are measured on a 5-point scale ranging from 'Never' to 'daily or almost daily'. The last two items from each scale are rated on a 3-point scale and are coded as 0 ('no'), 2 ('yes, but not in the last year') or 4 (yes, during the last year'). Risk for alcohol use disorders based on AUDIT scores are assigned as follows: a score of 0-7 indicates low risk; 8-15 indicates increasing risk; 16-19 indicates higher risk; and a score of 20 or greater indicates possible dependence. Risk for drug use disorders (in male populations where you would not expect drug users) are assigned as follows: a score of 1-5 indicates low risk, a score of 6 or greater indicates possible drug related problems (Berman et al., 2005). Internal consistency for the AUDIT and DUDIT were $\alpha$ = 0.88, and $\alpha$ = 0.85 respectively.

Prosocial behavior and total difficulties (as screening in the TD participants), emotional problems, peer problems, and hyperactivity (as measures of symptoms commonly co-occuring with CP) subscales of the *Strengths and Difficulties Questionnaire* (SDQ; Goodman 1997). The SDQ contains 25 items, rated on a 3-point scale from 'Not True' to 'Very True'. The SDQ has been extensively normed on a large-scale population of young people (e.g. Goodman 2001). The measures showed good internal consistency in our sample: Prosocial behaviour, $\alpha$ = 0.88; total difficulties, $\alpha$ = 0.93; emotional problems, $\alpha$ = 0.85; peer problems, $\alpha$ = 0.75; hyper-activity, $\alpha$ = 0.90.

## Appendix S2. Age and IQ Covariate Analysis - Model Specification:

**Model 1 – Social information use with age and IQ covariates:** Mixed model regression analyses were designed using the *lmer* function of the *lme4* package in R, version 4.0.0 (R Core Team, 2020). Model 1. tested whether social information use (*s*) was influenced by age, IQ, and group (conduct problems with high callous-unemotional (CP/HCU) traits, CP with low CU traits, typically developing (TD) coded as TD:0, CP/LCU:1, CP/HCU:2) as a fixed factor, and a subject level random intercept. The model was specified in R as follows:

s ~ age + IQ + group + (1|ID)

**Model 2 – Strategy use with Age and IQ covariates:** A logistic regression analysis model was designed using the *glmer* function of the *lme4* package in R, version 4.0.0. Model 2. tested whether participants' strategy use (coded as all-or-nothing; 1 or compromising: 0) was influenced by age, IQ and group (conduct problems with high callous-unemotional (CP/HCU) traits, CP with low CU traits (CP/LCU), typically developing (TD) coded as TD:0, CP/LCU:1, CP/HCU:2) as a fixed factor, and a subject level random intercept. The model was specified in R as follows:

extreme ~ group + age + IQ + (1|ID)

**Optimiser**: Bobyqa

## Appendix S3 Statistical Analyses of Participant Characteristics: Full Details

Groups (conduct problems with high callous-unemotional (CP/HCU) traits, CP with low CU traits (CP/LCU), typically developing (TD)) were matched for IQ (*F*(2, 113) =2.89, *p* = 0.06, η^2^ = 0.05). Groups differed significantly in age (*F*(2, 113)= 5.17, *p* = 0.007; η^2^ = 0.08), with the CP/LCU group being significantly younger than the CP/HCU group (post-hoc Bonferroni: *p* = 0.005).

Groups differed significantly on CP as measured by the CASI (*F*(2,113) *=* 90.58, *p*<.0001, η^2^ =0.62), with post-hoc comparisons using the Bonferroni HSD revealing that CP/HCU scored higher than CP/LCU (*p =* 0.002*)* and TD (*p*<.0001) groups, and CP/LCU higher than TD (*p*<.0001). Groups also differed significantly on CU traits as measured by the ICU (*F*(2, 113)=175.1, *p* <.0001; η^2^ = 0.76). Post-hoc comparisons using the Bonferroni correction indicated that the CP/HCU group scores were higher than CP/LCU (*p*<.0001), and TD (*p*<.0001) on the ICU. Scores for the CP/LCU group were higher than for the TD group (*p*<.0001).

Our measures of common co-occurring symptoms with CP revealed that groups did not differ on self-reported use of alcohol use (*χ*^2^ = 8.19, *p* = 0.08, φ_c_ = 0.19). Chi square tests revealed a significant group difference in drug use (*χ*^2^ = 6.34, *p* = 0.05, φ_c_ = 0.23). This significant effect did not hold when corrected for multiple comparisons using Bonforroni correction (all *p*s >0.5).

Groups differed on SDQ rated hyperactivity, with CP/HCU scoring higher than CP/LCU (*U* = 391, *p* = 0.03, *Â = 0.34*), and TD (*U* = 81.5, *p*<.0001, *Â* = *0.05*), and CP/LCU higher than TD (*U=*208.50*, p*<.0001, *Â = 0.13).*The CP/HCU and CP/LCU groups did not differ on emotional problems (*U* = 520, *p* = 0.47, *Â =*0.45), but both groups differed from TD (CP/HCU, *U* = 310, *p<*.0001, *Â =* 0.29; CP/LCU, *U=*349, *p*<.0001, *Â =* 0.22). Similarly, both CP groups differed from the TD group in peer problems (CP/HCU, *U* = 331, *p<*.0001, *Â =* 0.45; CP/LCU, *U=*360, *p*<.0001, *Â =* 0.22), but did not differ from each other (*U* = 5521.520, *p* = 0.49, *Â =*0.45).

ANOVA tests revealed that our groups differed significantly on screening measures for the TD group: SDQ rated prosocial behaviour (F(2, 111) =53.80, *p*<.0001, η^2^ =0.49) and total difficulties (F(2, 111) = 103.10, *p*<.001, η^2^ =0.65). Post-hoc Bonferroni tests revealed that the TD group scored significantly higher than both the CP/HCU (*p* <.0001) and LCU (*p* =0.0001) groups on prosocial behaviour, and that the CP/LCU group scored significantly higher than the HCU group (*p*<.0001). Post-hoc Bonferroni tests of the ANOVA of group scores on total difficulties revealed that the CP/HCU group scored significantly higher than both the TD (*p* <.0001) and LCU (*p* = 0.005) groups, and that the CP/LCU group scored significantly higher than the TD group (*p*<.0001). Two participants were removed from analysis of SDQ measures due to missing data.

## Appendix S4 Experimental Materials

The experimental task was completed on a laptop. Below are the task instruction text and screenshots one of experimental round, as seen by the participants. Stimuli were presented in a fixed order.

Instructions:

**Welcome.** In this task you have to make a number of estimates. With your estimates you can win points. The number of points you can win in this task depends on how accurate your estimates are. Click below to proceed to the task instructions.
<Continue>

This task consists of **5 rounds**. At the beginning of each round, you will observe an image showing a number of animals. For example: <image> The image will disappear after 6 seconds, upon which **you have to estimate how many animals were displayed. The more accurate your estimate, the more points you can earn.** We explain this later.

<Continue>

Once the image has disappeared, you have to enter your estimate of how many animals were displayed. This is your estimate for **part A** of a round. Once you have entered your estimate, **part B** of the round begins. You can observe the part A estimate of another participant. Over 100 people recently participated in this study and completed this task. In each round, you can observe the part A estimate of one of these previous participants. The previous participants saw the same image as you. They also saw it for 6 seconds. After the image disappeared, they also had to estimate how many animals were displayed. They could earn more points if their estimate was more accurate. You then have **to enter a second estimate.** You can enter the same estimate as in part A, or adjust it as you wish. This is your estimate for **part B** of a round. After that, the round is over and a new round begins. **If you have any questions, please ask the researcher now!**

<Continue>

**The more accurate your estimates, the more points you can earn in this task.** If you estimated the number of animals *exactly right*, you earn 100 points. For each animal that you are off, we subtract 5 points. Your points cannot become negative. For example, if the actual number of animals in an image was 60, and your estimate was 53, you were 7 off. This would mean that we subtract 7 x 5 = 35 points. **If you have any questions, please ask the researcher now!** Click 'Continue' if you understood your task. A brief quiz will follow to check your understanding.

<Continue>

To check your understanding of the task, please indicate for each of these statements whether they are correct or incorrect. In each round of this task you will view an image. You have to estimate how many animals were displayed in it. <Correct> <Incorrect>. Once you have entered your estimate, you can observe the estimate of another participant who completed this task before. You can then make a second estimate. <Correct> <Incorrect>. The more accurate your estimates, the more points you can earn. <Correct> <Incorrect>

<Continue>

When you click below, an image will appear showing a number of Ants. After **6 seconds**, the image disappears. A box will appear in which you have to estimate how many Ants there were. Click below when you are ready.

<Continue>

**AT END OF TASK:** You have now finished this task. Once this study has completed, we will inform you of the results. Thank you for your participation

## Appendix S5 Calibration of Social Information

Participants were informed that the social information seen on each round was the estimate of an adolescent participant at another school. In reality, if a participant’s *E_1_* was *lower* than the true value, *X* was 1.2 times *E_1_* (*X* = 1.2 $\cdot$ *E_1_*); if their *E_1_* was *higher* than the true value (the real number of animals on the screen), *X* was 0.8 times *E_1_* (*X* = 0.8 $\cdot$ *E_1_*). This setup allowed for a relatively constant scope for adjustment in each round, while experimentally controlling for possible effects of ‘distance weighting’, the observation that people tend to discount information that deviates too strongly from their initial estimate (Moussaïd et al., 2013). If *E_1_* was exactly correct (equal to the true value), a coin flip determined whether *X* on that round would be higher or lower. This ensured that *X* was sometimes higher and sometimes lower than the initial estimate. This minor deception was approved by the UCL ethics committee (project code: 0622/001).

## Appendix S6 Statistical Analyses

***Demographic Data***

Data for group matching and assessment of emotional and behavioural difficulties were analysed using one-way Analysis of Variance (or appropriate non-parametric equivalents) for continuous data and Chi-Squared tests for categorical data.

All analyses were carried out in R statistical software and R Studio (R version 4.0.0; Team, 2015). Tests and results are summarised in Table 1 and Table S1. Graphs of results (Fig 2) were produced using ggplot2 (Wikham, 2016), the table summarising mixed models (Table 2) was produced using sjPplot (Lüdecke et al., 2021). Mixed mdels were run using lmerTest (Kuznetsova et al., 2017).

***Experimental Data***

**Basic behavioural results**

Basic behavioural results were analysed using Analysis of Variance, comparing accuracy of initial estimate (*E_1_*/true value) between groups.

**Degree of social information use**

Regression analyses were carried out using Linear and Generalised Linear Mixed Effects models with the R package ‘lme4’ (Bates et al., 2015). For a full description of the models and suitability see S11.

Degree of social information use by group (CP/HCU, CP/LCU, TD) was assessed using a Linear Mixed Effects model (Table 2, Model 1), including group as a fixed effect, subject as a random effect, and social information use in the task rounds (*s*) as the outcome variable. Fixed effects were tested for difference from 0 using a Type II Wald Chi-Square test.

**Strategy when using social information**

Strategy use was assessed by examining how participants used social information to adjust estimates on individual rounds (Table 2, Model 2 of main manuscript). Strategies were classified as ‘compromising’ if participants’ second estimate fell between their initial estimate and the social information (0<*s*<1). Strategies were classified as all-or-nothing if participants second estimate was the same as their original estimate (*s*=0) or a direct copy of the social information (*s*=1). A Generalised Linear Mixed Effects model was used to compare strategy use between groups, including group as a fixed factor, subject as a random factor, and likelihood of all-or-nothing responding (1 = copy/stay response, 0 = compromising response) as the outcome variable. Fixed effects were tested for difference from 0 using a Type II Wald Chi-Square test.

## Appendix S7 Main Experimental Models: Specification and Assumption Checks

**Model 1 – Social information use:** Mixed model regression analyses were designed using the *lmer* function of the *lme4* package in R, version 4.0.0 (R Core Team, 2020). Model 1. tested whether social information use (*s*) was influenced by group (conduct problems with high callous-unemotional (CP/HCU) traits, CP with low CU traits, typically developing (TD) coded as TD:0, CP/LCU:1, CP/HCU:2) as a fixed factor, and a subject level random intercept. The model was specified in R as follows:

s ~ group + (1|ID)

**Model 2 – Strategy use:** A logistic regression analysis model was designed using the *glmer* function of the *lme4* package in R, version 4.0.0. Model 2. tested whether participants' strategy use (coded as all-or-nothing; 1 or compromising: 0) was influenced by group (conduct problems with high callous-unemotional (CP/HCU) traits, CP with low CU traits (CP/LCU), typically developing (TD) coded as TD:0, CP/LCU:1, CP/HCU:2) as a fixed factor, and a subject level random intercept. The model was specified in R as follows:

strategy use ~ group + (1 | ID), family=’binomial’)

Optimiser: ‘bobyqa’.

## Appendix S8 Additional covariate analysis

**Appendix S8a - Methods and statistics**

Cognitive and affective empathy were assessed using subscales of the Basic Empathy Scale (BES; Jolliffe & Farrington, 2006). This scale contains 20 items, scored on a five-point scale from ‘strongly disagree’ to ‘strongly agree’, nine items measuring cognitive empathy, eleven items measuring affective empathy. The cognitive and affective empathy subscales of the BES showed good internal reliability, with $\alpha$s of 0.701 and 0.713 respectively.

Cognitive perspective taking was assessed using the Interpersonal Reactivity Index perspective taking scale (IRI-PT; Davis, 1980). This scale contains seven items, scored on a five-point scale from ‘does not describe me well’ to ‘describes me very well’. The measure showed a fairly low reliability in our sample ($\alpha$ = 0.67).

**Appendix S8b – Model specification:**

**Social information use:** Three mixed model regression analyses were designed using the *lmer* function of the *lme4* package in R, version 4.0.0 (R Core Team, 2020). These models tested whether social information use (*s*) was influenced by group (conduct problems with high callous-unemotional (CP/HCU) traits, CP with low CU traits, typically developing (TD) coded as TD:0, CP/LCU:1, CP/HCU:2) as a fixed factor, and a subject level random intercept. Each model then included a covariate: model 1 – cognitive empathy, model 3 – affective empathy, model 5 – cognitive perspective taking. The models were specified in R as follows:

**Model 3:** s ~ group + cognitive empathy + (1|ID)

**Model 5:** s ~ group + affective empathy + (1|ID)

**Model 7:** s ~ group + affective perspective taking + (1|ID)

**Strategy use:** Three logistic regression analysis models were designed using the *glmer* function of the *lme4* package in R, version 4.0.0. Model 2. tested whether participants' strategy use (coded as all-or-nothing; 1 or compromising: 0) was influenced and group (conduct problems with high callous-unemotional (CP/HCU) traits, CP with low CU traits (CP/LCU), typically developing (TD) coded as TD:0, CP/LCU:1, CP/HCU:2) as a fixed factor, and a subject level random intercept. Each model then included a covariate: model 2 – cognitive empathy, model 4 – affective empathy, model 6 – cognitive perspective taking. The models were specified in R as follows:

**Model 4:** extreme ~ group + cognitive empathy + (1|ID)

**Model 6:** extreme ~ group + affective empathy + (1|ID)

**Model 8:** extreme ~ group + cognitive perspective taking + (1|ID)

**Optimiser**: Bobyqa

## Supplemental References

Babor, T. F., de la Fuente, J. R., Saunders, J., & Grant, M. (2001). The Alcohol Use Disorders Identification Test: Guidelines for use in. *Primary Care*.

Bates, D., Maechler, M., Bolker, B., & Walker, S. (2015). Fitting Linear Mixed-Effects Models Using lme4. *Journal of Statistical Software*, *67*(1), 1–48. https://doi.org/doi:10.18637/jss.v067.i01.

Berman, A. H., Bergman, H., Palmstierna, T., & Schlyter, F. (2005a). DUDIT manual the drug use disorders identification test. *London: Karolinska Institutet Department of Clinical Neuroscience*.

Berman, A. H., Bergman, H., Palmstierna, T., & Schlyter, F. (2005b). Evaluation of the Drug Use Disorders Identification Test (DUDIT) in criminal justice and detoxification settings and in a Swedish population sample. *European Addiction Research*, *11*(1), 22–31.

Davis, M. H. (1980). A multidimensional approach to individual differences in empathy. *Catalog of Selected Documents in Psychology*, *10*(85).

Essau, C. A., Sasagawa, S., & Frick, P. J. (2006). Callous-unemotional traits in a community sample of adolescents. *Assessment*, *13*(4), 454–469.

Gadow, K. D., & Sprafkin, J. (2005). Child and adolescent symptom inventory-4R. *Stony Brook, NY: Checkmate Plus*.

Goodman, R. (1997). *The Strengths and Difficulties Questionnaire: A research note. Child Psychology & Psychiatry & Allied Disciplines, 38 (5), 581-586*.

Goodman, R. (2001). Psychometric properties of the strengths and difficulties questionnaire. *Journal of the American Academy of Child & Adolescent Psychiatry*, *40*(11), 1337–1345.

Jolliffe, D., & Farrington, D. P. (2006). Development and validation of the Basic Empathy Scale. *Journal of Adolescence*, *29*(4), 589–611. https://doi.org/10.1016/j.adolescence.2005.08.010

Kuznetsova, A., Brockhoff, P. B., & Rune, H. B. (2017). *Tests in Linear Mixed Effects Models*. *82*.

Lüdecke, D., Bartel, A., Schwemmer, C., Powell, C., Djalovski, A., & Titz, J. (2021). *sjPlot: Data Visualization for Statistics in Social Science* (2.8.9) [Computer software]. https://CRAN.R-project.org/package=sjPlot

Moussaïd, M., Kämmer, J. E., Analytis, P. P., & Neth, H. (2013). Social Influence and the Collective Dynamics of Opinion Formation. *PLOS ONE*, *8*(11), e78433. https://doi.org/10.1371/journal.pone.0078433

R Core Team. (2020). *R: A language and environment for statistical computing.* R Foundation for Statistical Computing, Vienna, Austria. https://www.R-project.org/.

Schielzeth, H., Dingemanse, N. J., Nakagawa, S., Westneat, D. F., Allegue, H., Teplitsky, C., Réale, D., Dochtermann, N. A., Garamszegi, L. Z., & Araya‐Ajoy, Y. G. (2020). Robustness of linear mixed-effects models to violations of distributional assumptions. *Methods in Ecology and Evolution*, *11*(9), 1141–1152. https://doi.org/10.1111/2041-210X.13434

Team, Rs. (2015). RStudio: Integrated development for R. RStudio. *Inc., Boston, MA*, *700*.

Wikham, H. (2016). *ggplot2: Elegant Graphics for Data Analysis* [R]. ttps://ggplot2.tidyverse.org
